# Mediation of the effect of malaria in pregnancy on stillbirth and neonatal death in an area of low transmission: observational data analysis

**DOI:** 10.1186/s12916-017-0863-z

**Published:** 2017-05-10

**Authors:** Kerryn A. Moore, Freya J. I. Fowkes, Jacher Wiladphaingern, Nan San Wai, Moo Kho Paw, Mupawjay Pimanpanarak, Verena I. Carrara, Jathee Raksuansak, Julie A. Simpson, Nicholas J. White, François Nosten, Rose McGready

**Affiliations:** 1 Centre for Epidemiology and Biostatistics, Melbourne School of Population and Global Health, The University of Melbourne, Melbourne, VIC Australia; 2 Macfarlane Burnet Institute for Medical Research and Public Health, Melbourne, VIC Australia; 3 Department of Epidemiology and Preventive Medicine and Department of Infectious Diseases, Monash University, Melbourne, VIC Australia; 4 Shoklo Malaria Research Unit, Mahidol-Oxford Tropical Medicine Research Unit, Faculty of Tropical Medicine, Mahidol University, Mae Sot, Thailand; 5 Mahidol-Oxford Tropical Medicine Research Unit, Faculty of Tropical Medicine, Mahidol University, Bangkok, Thailand; 6 Centre for Tropical Medicine and Global Health, Nuffield Department of Medicine, University of Oxford, Oxford, UK

**Keywords:** Malaria in pregnancy, Stillbirth, Neonatal death, Mediation analysis

## Abstract

**Background:**

Malaria in pregnancy is preventable and contributes significantly to the estimated 5.5 million stillbirths and neonatal deaths that occur annually. The contribution of malaria in pregnancy in areas of low transmission has not been quantified, and the roles of maternal anaemia, small-for-gestational-age status, and preterm birth in mediating the effect of malaria in pregnancy on stillbirth and neonatal death are poorly elucidated.

**Methods:**

We analysed observational data routinely collected at antenatal clinics on the Thai-Myanmar border (1986–2015). We used Cox regression and sequential mediation analysis to determine the effect of falciparum and vivax malaria in pregnancy on antepartum (death *in utero*) and intrapartum (death during labour) stillbirth and neonatal mortality as well as mediation through maternal anaemia, preterm birth, and small-for-gestational-age status.

**Results:**

Of 61,836 women, 9350 (15%) had malaria in pregnancy, and 526 (0.8%) had stillbirths. In a sub-set of 9090 live born singletons followed from birth there were 153 (1.7%) neonatal deaths. The hazard of antepartum stillbirth increased 2.24-fold [95% confidence interval: 1.47, 3.41] following falciparum malaria (42% mediated through small-for-gestational-age status and anaemia), driven by symptomatic falciparum malaria (hazard ratio, HR: 2.99 [1.83, 4.89]) rather than asymptomatic falciparum malaria (HR: 1.35 [0.61, 2.96]). The hazard of antepartum stillbirth increased 2.21-fold [1.12, 4.33] following symptomatic vivax malaria (24% mediated through small-for-gestational-age status and anaemia) but not asymptomatic vivax malaria (HR: 0.54 [0.20, 1.45]). There was no association between falciparum or vivax malaria in pregnancy and intrapartum stillbirth (falciparum HR: 1.03 [0.58, 1.83]; vivax HR: 1.18 [0.66, 2.11]). Falciparum and vivax malaria in pregnancy increased the hazard of neonatal death 2.55-fold [1.54, 4.22] and 1.98-fold [1.10, 3.57], respectively (40% and 50%, respectively, mediated through small-for-gestational-age status and preterm birth).

**Conclusions:**

Prevention of malaria in pregnancy, new and existing interventions to prevent small-for-gestational-age status and maternal anaemia, and improved capacity for managing preterm and small-for-gestational-age newborns will reduce the number of malaria-associated stillbirths and neonatal deaths in malaria-endemic areas.

**Electronic supplementary material:**

The online version of this article (doi:10.1186/s12916-017-0863-z) contains supplementary material, which is available to authorized users.

## Background

Progress in reducing the 2.6 million stillbirths and 2.9 million neonatal deaths that occur each year, of which 98% are in resource-limited settings and often from preventable causes, requires accurate estimation of contributing factors [1, 2]. Falciparum malaria in pregnancy is a preventable cause of stillbirth and neonatal death [3]. Annually, 125 million women are at risk of malaria in pregnancy, and 19.7% of stillbirths in Africa are attributed to falciparum malaria in pregnancy [3, 4]. Malaria in pregnancy is also associated with maternal anaemia and small-for-gestational-age (SGA) babies, which are risk factors for stillbirth and preterm birth. Preterm birth remains the leading cause of neonatal death [5].

Falciparum malaria in pregnancy has been recognised as a cause of stillbirth since the early twentieth century [6], but no studies with accurate gestational age assessment have differentiated between antepartum (prelabour death *in utero*) and intrapartum (death during labour) stillbirth or the gestation time of malaria detection [7]. The effects of malaria in pregnancy on antepartum stillbirth and intrapartum stillbirth are likely to be different, and the relative contribution of antepartum and intrapartum stillbirth to total stillbirths varies between populations [8]. Additionally, the contribution of mediating factors between malaria in pregnancy and stillbirth and neonatal death, including maternal anaemia, SGA status, and preterm birth have not been fully elucidated, preventing identification of potential pathways that can be targeted by existing or new interventions.

The effect of malaria may be greater in areas of low transmission where there is little or no maternal immunity, but most studies of malaria in pregnancy and stillbirth or neonatal death are from areas of high transmission in Africa. The current estimate of the number of stillbirths attributed to malaria in pregnancy is specific to falciparum malaria in Africa [9]. Few studies (with conflicting results) have assessed the effect of vivax malaria in pregnancy on stillbirth or neonatal death [10–13]. The majority of stillbirths and neonatal deaths occur outside of Africa where malaria transmission is low and falciparum and vivax malaria coexist, and the epidemiological shift from high to low transmission demands estimates from low transmission settings [14]. In *The Lancet* 2011 Stillbirth Series, the effect of malaria in pregnancy and anaemia were two of the five top-ranked options for advancing epidemiological understanding of stillbirth, and from the 2016 Series there were still inadequate data for risk factor analysis, especially for regions outside of Africa [3].

This paper extends a previous analysis of the effect of malaria and artemisinin treatment in the first trimester of pregnancy and miscarriage, which found a strong association between first-trimester malaria (either falciparum or vivax) and miscarriage, especially following recurrence [15]. The novelty of this observational data analysis lies in assessment of the effects of both falciparum and vivax malaria in pregnancy across the entire range of pregnancy loss including miscarriage, antepartum stillbirth, intrapartum stillbirth, and neonatal mortality in an area of low transmission, and the exploration of mediation through maternal anaemia, SGA, and preterm birth.

## Methods

### Catchment area and population

Since 1986, the Shoklo Malaria Research Unit (SMRU) has collected data on prospectively followed pregnant women attending antenatal clinics (ANCs) on the Thai-Myanmar border, including confirmed *Plasmodium* spp. infections and pregnancy outcomes. In the refugee camps where the denominator is known, 90% of pregnant women in the population attend SMRU ANCs. Syphilis and HIV were not routinely tested for, but prevalence is very low [16]. The Oxford Tropical Research Ethics Committee granted ethical approval for audits of SMRU clinical records (OXTREC 28-09), and the Tak Community Advisory Board granted local approval (TCAB-4/1/2015).

### Procedures

This is an analysis of prospective observational data collected at SMRU ANCs since 1986. Women were encouraged to attend antenatal care early and return weekly throughout their pregnancy for malaria screening (finger-prick blood sample examined by trained microscopists) because there were no suitable preventive interventions for malaria in this region [17, 18]. With each positive screen, information on species, symptoms, fetal viability, and gestation were recorded. Malaria was defined as the presence of asexual parasites in the peripheral blood (per 500 leucocytes or 1000 erythrocytes). Consecutive positive slides of the same species less than 7 days apart were counted as one episode. Symptomatic malaria was defined as parasitaemia plus a temperature ≥37.5 °C or a history of fever in the past 48 hours. Vivax malaria was treated with chloroquine. Falciparum malaria was treated with quinine in all trimesters until 1995 and thereafter with quinine in the first trimester and artemisinin-based treatments in the second and third trimesters (and in the first trimester for cases of severe malaria or hyperparasitaemia [>4% infected red blood cells]).

Haematocrit was measured fortnightly, and maternal anaemia (haematocrit <30%) was treated with ferrous sulphate and folic acid. Gestational age was predominantly estimated by fundal height measurement (1986–1994), the Dubowitz Gestational Age Assessment (1992–2002), and ultrasound biometry (2001–present) [7]. The gestational age used to differentiate stillbirth from miscarriage in high-income settings ranges from 20 to 24 weeks. However, at SMRU neonatal intubation and ventilator support are unavailable, and only 37% of newborns delivered between 24 and less than 28 weeks’ gestation were live born, of which 98% died within 28 days [19]. Therefore, SMRU defines stillbirth as a baby born dead from 28 weeks’ gestation, in accordance with the World Health Organisation (WHO) and *The Lancet* 2016 Stillbirth Series [1], and a miscarriage as fetal demise before 28 weeks’ gestation. Intrauterine fetal demise was confirmed by ultrasound since 2001, or prior to 2001, by the absence of a fetal heartbeat by Pinard horn or hand-held Doppler monitor, loss of fetal movement, or reduced symphysis fundal height measurement. SGA, a proxy for fetal growth, was defined as a birthweight for gestational age below the 10th centile from INTERGROWTH-21st standards; birthweight for gestational age could only be determined for newborns with a gestational age ≥24 weeks and a birthweight measured within 72 hours of birth [20]. Birthweight for gestational age was considered unknown if a fetal heartbeat was not recorded within 7 days of birth to avoid measurement error in fetuses who may have died some time before birth. Preterm birth was defined as birth before 37 weeks’ gestation. Neonatal death was defined as death of a liveborn of at least 28 weeks’ gestation in the first 28 days of life.

Primary exposures were malaria in pregnancy (detected at any time during pregnancy) and the trimester of last malaria detection. Trimester cut-offs were 14 weeks’ and 28 weeks’ gestation. The primary outcome was stillbirth. Stillbirths were retrospectively classified by reviewing antenatal and delivery records as antepartum (prelabour fetal demise *in utero*) or intrapartum (fetal demise during labour). Secondary outcomes were fetal loss at any time (both miscarriage and stillbirth) and neonatal death. For the analysis of stillbirth and fetal loss, we included women with a singleton pregnancy and an estimated gestational age. For the analysis of neonatal mortality, we used data from all SMRU cohort studies that intended to follow newborns for at least the first 28 days of life; details of these studies are given in Additional file 1. Only a sub-set of mother-newborn pairs who attend SMRU ANCs were also enrolled in cohort studies.

### Statistical analysis

**Fig. 1:**
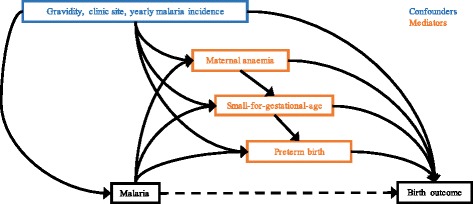
Directed acyclic graph for the mediated association between malaria in pregnancy and birth outcome. Malaria was either falciparum or vivax, and birth outcome was antepartum stillbirth or neonatal death, depending on the association being assessed for mediation. Preterm birth was only included in models where neonatal death was the outcome. Maternal anaemia was only included in models where stillbirth was the outcome because <1% of women in the neonatal death sub-set were anaemic

Multivariable Cox proportional hazards models were used to assess the association between malaria during pregnancy and stillbirth and fetal loss, with gestation as the time scale and censoring at the time of death or time last seen. Cox regression was chosen to account for variable follow-up times, left truncation, loss to follow-up, time-varying malaria status, and time-varying risk of stillbirth, fetal loss, and neonatal death (see Additional file 2). Live birth, antepartum stillbirth, and intrapartum stillbirth were competing risks, as experiencing one precludes observation of another. Cox models were also used to assess the association between malaria during pregnancy and neonatal mortality, with days since birth as the time scale, and censoring at the time of death, time last seen, or 28 days. All models were adjusted for gravidity, clinic site (refugee camp or migrant community), and yearly malaria incidence (see Additional file 3). When differentiating between species, women with both vivax and falciparum malaria (either a mixed infection or multiple infections of different species) were not analysed. Likewise, when differentiating between asymptomatic and symptomatic malaria, women with both asymptomatic and symptomatic episodes were not analysed. When malaria is not prefaced with ‘asymptomatic’ or ‘symptomatic’, the analysis was performed without differentiating between asymptomatic and symptomatic episodes. We assumed non-informative right censoring, and we did not test the non-proportional hazards assumption of Cox regression because this becomes non-interpretable with a time-varying exposure. The population proportions of stillbirths and neonatal deaths attributed to malaria were estimated [21]. Where associations were observed between malaria and stillbirth or neonatal death, we performed sequential mediation analyses to estimate marginal natural direct and indirect effects mediated through SGA and maternal anaemia (and preterm birth in the case of neonatal death) by calculating potential outcomes from logistic models with inverse probability weighting to achieve balance in the malaria groups in terms of the confounders (gravidity, clinic site, and yearly malaria incidence) (Fig. 1; see Additional file 4) [22]. All analyses were performed in Stata version 13 (StataCorp, College Station, TX, USA).

## Results

**Fig. 2:**
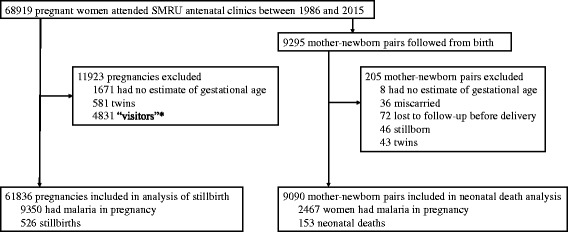
Analysis profile. ‘Visitors’, who are not residents of the migrant communities or refugee camps where SMRU clinics are located, were excluded because they do not regularly attend SMRU clinics throughout their pregnancy

Between 6 January 1986 and 31 December 2015, 68,919 pregnant women presented to SMRU ANCs, of whom 61,836 had a singleton pregnancy and an estimated gestational age (Fig. 2). Of these women, half presented to the ANC during the first trimester, and the median number of consultations was 11 (Table 1). There were 9350 women (15%) who had at least one episode of malaria in pregnancy, and they were more likely to be younger, primigravid, and live in a migrant community compared to women with no malaria in pregnancy (all *p* < 0.001; Table 1). Fetal loss occurred in 5850 pregnancies (9.5%), of which 526 were stillbirths (9%) [49% (260/526) antepartum; 34% (178/526) intrapartum; and 17% (88/526) uncertain] and 5324 (91%) were miscarriages. The overall stillbirth rate was 10.1/1000 live births (95% confidence interval, CI: 9.2, 11.0; 526/52,194 births). The last malaria episode detected during pregnancy (either falciparum or vivax) was more likely to be symptomatic if it was detected during the first trimester rather than the second or third trimester (see Additional file 5). The proportion of women lost to follow-up and the median gestation time when last seen were similar between women with and without malaria during pregnancy (1389 [15%] at median gestation week 27.5 and 8173 [16%] at median gestation time 28.3, respectively).

**Table 1:** Cohort demographics, *N* = 61,836

Variable	No malaria, *N* = 52,486	Malaria, *N* = 9350
EGA at first ANC (start of follow-up), weeks	14.3 {9.0, 22.7}, 0–43.0	13.7 {9.0, 21.1}, 0–41.1
First ANC during 1st trimester	25,548 (49)	4753 (51)
Number of ANC consultations	10 {4, 19}, 1–43	12 {5, 21}, 1–42
EGA method		
Ultrasound biometry	33,443 (64)	4793 (51)
Dubowitz	8745 (17)	1517 (16)
Fundal height	6337 (12)	2126 (23)
Last menstrual period	3961 (8)	914 (10)
Maternal age, years	26 {21, 31}, 13–53	25 {20, 30}, 13–51
Primigravid^a^	13756 (26)	2746 (29)
Haematocrit^a,b^, %	34 {31, 36}, 8–53	32 {30, 35}, 10–48
Anaemia^a,b^	4817 (10)	1711 (21)
Current smoker^a^	9765 (22)	2232 (34)
Site		
Refugee camp	32,689 (62)	4369 (47)
Migrant community	19,797 (38)	4981 (53)
Malaria in pregnancy	–	9350 (100)
Falciparum malaria only	–	3478 (37)
Last detected in first trimester	–	715 (21)
Last detected in second trimester	–	1411 (41)
Last detected in third trimester	–	1352 (39)
Recurrent falciparum malaria	–	892 (26)
Vivax malaria only	–	4274 (46)
Last detected in first trimester	–	654 (15)
Last detected in second trimester	–	1365 (32)
Last detected in third trimester	–	2255 (53)
Recurrent vivax malaria	–	1470 (34)
Falciparum and vivax malaria^c^	–	1598 (17)
Fetal loss	5246 (10)	604 (6)
Small-for-gestational-age^a,d^	7514 (20)	1870 (27)
Preterm birth^d^	3318 (9)	831 (11)
Caesarean section^d^	1467 (4)	158 (2)
Place of delivery^d^		
SMRU delivery unit	23,546 (67)	2941 (47)
At home	8534 (24)	2778 (44)
Hospital	3016 (9)	570 (9)
Stillbirth (all)	422 (1.0)	104 (1.3)
Antepartum stillbirth	202 (0.5)	58 (0.7)
Intrapartum stillbirth	145 (0.3)	33 (0.4)
Unclassified stillbirth^e^	75 (0.2)	13 (0.2)
Lost to follow-up^f^	8173 (16)	1389 (15)
EGA when last seen, weeks’ gestation	28.3 {18.6, 35.5}, 4–47	27.5 {19.0, 34.5}, 4–42

*EGA* estimated gestational age, *ANC* antenatal clinic, *IQR* interquartile range. Numbers are median {IQR}, range, or frequency (%)

^a^Missing data: gravidity 108 [2%] (79 [2%] with no malaria; 29 [0%] with malaria); smoking status 11,733 [19%] (8893 [17%] with no malaria; 2840 [30%] with malaria); haematocrit 7173 [12%] (5798 [11%] with no malaria; 1375 [32%] with malaria); place of delivery 15,698 [25%] (13,020 [25%] with no malaria; 2678 [29%] with malaria); caesarean section 1629 [3%] (1470 [3%] with no malaria; 159 [2%] with malaria); small-for-gestational-age 7308 [14%] (6140 [14%] with no malaria; 1168 [14%] with malaria)

^b^Last measurement during pregnancy; anaemia defined as last haematocrit measurement during pregnancy <30%; only 0.16% of women were severely anaemic (haematocrit <20%)

^c^Women with either a mixed infection or multiple infections of different species

^d^In women who gave birth after 28 weeks’ gestation

^e^Time of fetal demise (i.e. antepartum or intrapartum) unknown

^f^Women lost to follow-up were more likely to be primigravid, smokers, migrant, and anaemic

### The association between malaria in pregnancy and stillbirth

Women were at risk of stillbirth and under observation for a total of 508,092 pregnancy-weeks, and there were 0.51 (95% CI: 0.45, 0.58) antepartum stillbirths (*N* = 260) and 0.35 (95% CI: 0.30, 0.40) intrapartum stillbirths per 1000 pregnancy-weeks (*N* = 178). Falciparum malaria in pregnancy increased the hazard of all stillbirths 1.69-fold (95% CI: 1.25, 2.29; *p* = 0.001), but vivax malaria did not (hazard ratio, HR = 1.03; 95% CI: 0.70, 1.50; *p* = 0.892), and 4.5% (95% CI: 2.4, 6.5) of all stillbirths were attributed to falciparum malaria. The stillbirth rate was 9.5/1000 births (95% CI: 8.6, 10.5) in women with no malaria in pregnancy and 13.1 (95% CI: 10.7, 15.8) in women with malaria in pregnancy.

**Fig. 3:**
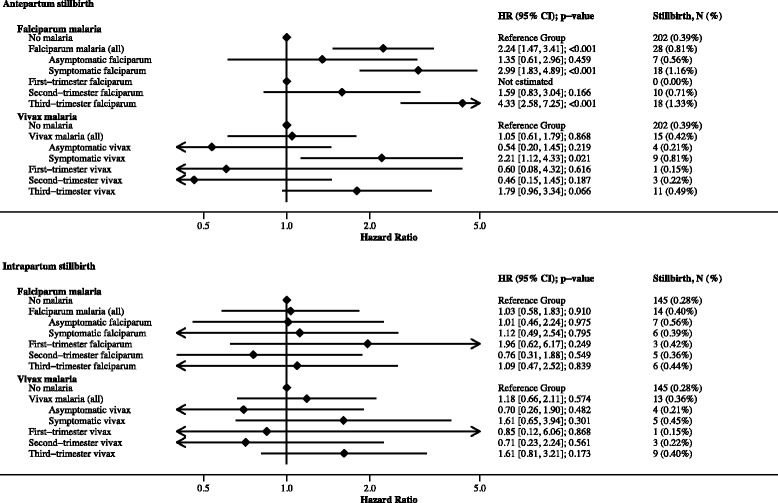
The association between falciparum and vivax malaria in pregnancy and antepartum or intrapartum stillbirth. The reference group refers to women without falciparum malaria or vivax malaria in pregnancy. Models include women lost to follow-up (until gestation time last seen), but percentage calculations for stillbirth do not. Where the numbers of stillbirths in the asymptomatic and symptomatic malaria categories do not total the number of stillbirths in the malaria (all) category, missing values for the presence of symptoms should be assumed. First-, second-, and third-trimester malaria refers to both symptomatic and asymptomatic malaria; in women with multiple episodes during pregnancy the trimester categorisation is based on the last episode detected. Associations were similar when the analysis was restricted to women with only one episode of malaria in pregnancy (see Additional file 6). Models were adjusted for gravidity, clinic site, and yearly malaria incidence. See Additional file 7 for table versions of this figure, including univariable associations

Symptomatic falciparum malaria, and to a much lesser extent asymptomatic falciparum malaria, increased the hazard of antepartum stillbirth 2.99-fold (95% CI: 1.83, 4.89; *p* < 0.001) and 1.35-fold (95% CI: 0.61, 2.96; *p* = 0.459), respectively (Fig. 3). The association between falciparum malaria and antepartum stillbirth was strongest in women whose last episode was detected during the third trimester (HR: 4.33; 95% CI: 2.58, 7.25; *p* < 0.001), rather than the second trimester (HR: 1.59; 95% CI: 0.83, 3.04; *p* = 0.166) or first trimester (zero stillbirths in women with first-trimester malaria only) (Fig. 3). The proportion of antepartum stillbirths attributed to falciparum malaria was 6.7% (95% CI: 4.4, 9.0). Symptomatic vivax malaria or vivax malaria last detected and treated in the third trimester increased the hazard of antepartum stillbirth 2.21-fold (95% CI: 1.12, 4.33; *p* = 0.021) and 1.79-fold (95% CI: 0.96, 3.34; *p* = 0.066), respectively, but asymptomatic vivax malaria did not (HR: 0.54; 95% CI: 0.20, 1.45; *p* = 0.219) (Fig. 3). There was no association between falciparum or vivax malaria and intrapartum stillbirth (falciparum HR: 1.03 [0.58, 1.83]; vivax HR: 1.18 [0.66, 2.11]) (Fig. 3). Cases of recurrent malaria did not influence the association between malaria in pregnancy and antepartum stillbirth (see Additional file 6). Fig. 3 and its tabular version, Additional file 7, detail the association between malaria and stillbirth. Mediation analyses showed that 42% and 24% of the effect of falciparum malaria on antepartum stillbirth and symptomatic vivax malaria on antepartum stillbirth, respectively, was mediated through SGA and maternal anaemia (Table 2).

**Table 2:** Mediation of the association between malaria in pregnancy and antepartum stillbirth or neonatal death

Mediating variable/s	Natural indirect effect, risk ratio (95% CI)	Natural direct effect, risk ratio (95% CI)	Proportion indirect
Falciparum malaria and antepartum stillbirth			
SGA only	1.09 (1.05, 1.14)	1.61 (0.84, 2.67)	18%
Maternal anaemia and SGA	1.23 (1.15, 1.34)	1.48 (0.77, 2.46)	42%
Symptomatic vivax malaria and antepartum stillbirth			
SGA only	1.10 (1.04, 1.17)	1.91 (0.65, 3.49)	16%
Maternal anaemia and SGA	1.13 (1.06, 1.23)	1.73 (0.59, 3.20)	24%
Falciparum malaria and neonatal death			
Preterm birth only	1.25 (1.10, 1.43)	2.27 (1.29, 3.63)	26%
SGA and preterm birth	1.30 (1.15, 1.49)	1.82 (1.07, 2.89)	40%
Vivax malaria and neonatal death			
Preterm birth only	1.20 (1.05, 1.37)	1.58 (0.94, 2.44)	31%
SGA and preterm birth	1.25 (1.09, 1.44)	1.32 (0.81, 1.98)	50%

Numbers are risk ratios (95% confidence interval). Natural direct effect (*NDE*, the effect of malaria on birth outcome, not mediated through the specified mediator/s); natural indirect effect (*NIE*, the effect of malaria on birth outcome, mediated through the specified mediator/s); *SGA* small-for-gestational-age. Note that total effects (NDE + NIE) from mediation analysis are not equivalent to the magnitude of associations obtained from Cox regression, which accounts for lost to follow-up and time-varying exposure and risk, because methods that incorporate time-to-event data in mediation analyses are limited. Maternal anaemia was defined as a final haematocrit during pregnancy of <30% (measured fortnightly throughout pregnancy). SGA was defined as a birthweight for gestational age below the 10th centile of INTERGROWTH-21st centiles. Maternal anaemia and SGA were included as binary variables because the association between the continuous variables, haematocrit and birthweight-for-gestational-age Z-score, and log_e_(odds) of stillbirth and/or neonatal death was non-linear. Maternal anaemia was not considered as a mediator of the association between malaria in pregnancy and neonatal death because <1% of women in this sub-set were anaemic. For further details on calculation and formal definitions of NDEs and NIEs, see Additional file 4.

### The association between malaria in pregnancy and fetal loss

**Fig. 4:**
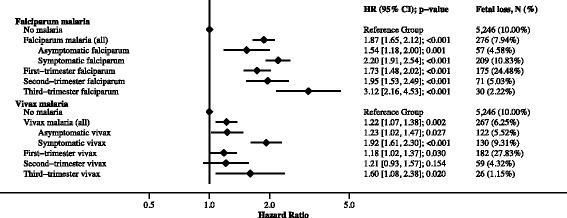
The association between falciparum and vivax malaria in pregnancy and fetal loss. The reference group refers to women without falciparum malaria or vivax malaria in pregnancy. Models include women lost to follow-up (until gestation time last seen), but percentage calculations for fetal loss do not. Where the numbers of fetal losses in the asymptomatic and symptomatic malaria categories do not total the number of stillbirths in the malaria (all) category, missing values for the presence of symptoms should be assumed. First-, second-, and third-trimester malaria refers to both symptomatic and asymptomatic malaria; in women with multiple episodes during pregnancy the trimester categorisation is based on the last episode detected. Models were adjusted for gravidity, clinic site, and yearly malaria incidence. See Additional file 8 for a table version of this figure, including univariable associations

Women were at risk of fetal loss and under observation for a total of 1,135,949 pregnancy-weeks, and there were 5.14 (95% CI: 5.01, 5.28) fetal losses per 1000 pregnancy-weeks (*N* = 5850); the rate of fetal loss was highest very early in pregnancy (see Additional file 2). Asymptomatic and symptomatic falciparum malaria were both strongly associated with fetal loss (HRs: 1.54 [1.18, 2.00; *p* < 0.001] and 2.20 [1.91, 2.54; *p* < 0.001], respectively). Falciparum malaria detected and treated in the first, second, or third trimester was strongly associated with fetal loss (HRs: 1.73 [95% CI: 1.48, 2.02; *p* < 0.001], 1.95 [95% CI: 1.53, 2.49; *p* < 0.001], 3.12 [95% CI: 2.16, 4.53; *p* < 0.001], respectively). See Fig. 4 and Additional file 8. Asymptomatic vivax malaria was moderately associated with fetal loss (HR: 1.23 [1.02, 1.47; *p* = 0.027], while symptomatic vivax malaria was strongly associated with fetal loss (HR: 1.92 [1.61, 2.30; *p* < 0.001]). Vivax malaria detected and treated in the first, second, or third trimester was moderately associated with fetal loss (HRs: 1.18 [95% CI: 1.02, 1.37; *p* = 0.030], 1.21 [95% CI: 0.93, 1.57; *p* = 0.154], 1.60 [95% CI: 1.08, 2.38; *p* = 0.020], respectively) (Fig. 4). Associations between falciparum and vivax malaria in pregnancy and fetal loss were greater following recurrent malaria; this effect was driven by strong associations between recurrent malaria and miscarriage rather than stillbirth (see Additional file 6).

### The association between malaria in pregnancy and neonatal mortality

Between 1993 and 2013, 9295 newborns were enrolled in SMRU birth cohorts (see Additional file 1), from which there were 9090 live born singletons with an estimated gestational age available for this analysis (Fig. 2). Cohort demographics of this sub-set of mother-newborn pairs were similar to those of the entire SMRU ANC cohort, except that <1% of women were anaemic (see Additional file 9). Neonates were at risk of neonatal death and under observation for a total of 6292 neonate-months, and there were 0.024 (95% CI: 0.021, 0.028) neonatal deaths per neonate-month (*N* = 153; 1.7%). The proportion of neonates lost to follow-up before 28 days was slightly higher in neonates of mothers with malaria during pregnancy (39%) than without malaria during pregnancy (31%), but the median time when last seen was on the day of birth for both groups (Additional file 9). Falciparum and vivax malaria during pregnancy were associated with a 2.55-fold (95% CI: 1.54, 4.22; *p* < 0.001; symptomatic HR: 2.85 [1.65, 4.93; *p* < 0.001]; asymptomatic HR: 1.68 [0.52, 5.48; *p* = 0.387]) and 1.98-fold (95% CI: 1.10, 3.57; *p* = 0.023; symptomatic HR: 2.39 [0.95, 5.97; *p* = 0.063]; asymptomatic HR: 2.35 [1.12, 4.95; *p* = 0.024]) increase in the hazard of neonatal death, respectively. The proportion of neonatal deaths attributed to falciparum and vivax malaria was 11.3% (95% CI: 7.5, 14.9) and 7.9% (95% CI: 3.0, 12.5), respectively. Mediation analyses showed that 40% and 50% of the effect of falciparum malaria on neonatal death and vivax malaria on neonatal death, respectively, was mediated through preterm birth and SGA (Table 2).

## Discussion

In this analysis of prospective observational data, we found that treated falciparum malaria was associated with antepartum but not intrapartum stillbirth, especially if symptomatic or detected later in pregnancy. Falciparum and vivax malaria in pregnancy, regardless of how early they were detected and treated, were associated with fetal loss, and treated falciparum and vivax malaria in pregnancy were associated with neonatal death.

A major strength of this analysis was the prospective follow-up of pregnant women who present early to antenatal care, so that gestational age could be accurately assessed and detailed data on malaria in pregnancy could be collected. Malaria in pregnancy was associated with antepartum stillbirth but not intrapartum stillbirth, so the contribution of malaria in pregnancy will vary according to the relative contribution of antepartum and intrapartum stillbirths to total stillbirths in a given population. Combining antepartum and intrapartum stillbirths will also attenuate the overall effect of malaria in pregnancy on stillbirth, and may contribute to the erroneous conclusion that malaria does not contribute to stillbirth [23].

Although many women in our analysis had recurrent malaria (either novel, recrudescent, or relapse in the case of vivax), the associations between malaria in pregnancy and antepartum stillbirth did not increase following recurrent malaria. This indicates that associations with stillbirth were not influenced by cases of recurrent malaria, which were more common in women whose last malaria episode was detected in the third trimester and in women with vivax malaria. In a previous analysis of these data, we found that the association between first-trimester malaria (either falciparum or vivax) and miscarriage was greater following a recurrent episode [15]. In women with recurrent malaria, the trimester of malaria detection pertained to the last malaria episode; this may have underestimated an effect of malaria in the first or second trimester.

Although we accounted for sources of bias including left truncation, time-varying exposures and risks, and confounding variables using multivariable Cox regression, we cannot rule out potential bias due to time or differential misclassification of stillbirths as miscarriages (or vice versa), as data were collected over 30 years and methods of estimating gestational age and confirming fetal loss have changed (discussed further in Additional file 10). Misclassification of stillbirths as antepartum or intrapartum was also possible, since this was determined retrospectively from antenatal and delivery records; however, when the time of fetal demise was unknown, stillbirths were categorised as ‘unclassified’ (17% of all stillbirths) rather than as either antepartum or intrapartum. Further, we cannot rule out the potential confounding effects of unmeasured variables (most notably smoking and detailed data on maternal demographics) needed to infer causal effects when analysing observational data (see Additional file 3). Many of our confidence intervals were also wide due to the rarity of stillbirth and neonatal death. The rate of stillbirth is low relative to those of other resource-limited settings, despite efforts to ascertain birth outcomes in all women (including those who delivered at home), and may be due in part to the low incidence of syphilis, the high number of ANC consultations, and the high proportion of deliveries attended by a skilled birth attendant at SMRU clinics [16, 24, 25]. The proportion of women lost to follow-up was not negligible, and we assumed non-informative right censoring (whereby the loss to follow-up is not due to experiencing the outcome) (see Additional file 10). Of particular note, we cannot differentiate the effect of maternal factors during pregnancy on the fetus and the environment of the neonate (e.g. local malaria transmission, socio-economic factors, etc.), which may have confounded the association between malaria in pregnancy and neonatal death.

In *The Lancet* 2016 Stillbirth Series, 8% of stillbirths worldwide (208,906) were estimated to be attributed to falciparum malaria in pregnancy, but this estimate only considered the effect of falciparum malaria on stillbirth in Africa because of limitations in available risk and prevalence data [3]. However, most stillbirths occur in regions outside of Africa where malaria transmission is low (and falciparum and vivax coexist) [24], and the effect of malaria on stillbirth is likely to be greater in areas of low transmission where there is little or no maternal immunity [10, 14]. In this area of low transmission, but in the context of early detection and treatment, we found that falciparum malaria detected during pregnancy more than doubled the risk of antepartum stillbirth, and 4.5% of all stillbirths in our population were attributed to falciparum malaria. Therefore, in addition to the 208,906 stillbirths attributed to falciparum malaria in pregnancy in Africa, it is likely that tens of thousands of stillbirths outside of Africa could also be attributed to malaria in pregnancy; this should not be ignored.

Our results substantiate previous observations from small studies [10–12] that vivax malaria may be associated with stillbirth; symptomatic vivax malaria doubled the risk of antepartum stillbirth. This association was mediated through SGA and maternal anaemia. Cytokines and fever may also be implicated, which would also explain the difference in the effect of falciparum malaria between symptomatic and asymptomatic women. The association between falciparum malaria and stillbirth was still present (though weaker and no longer statistically significant) when restricted to asymptomatic falciparum malaria. In Uganda, falciparum malaria detected at delivery, even at sub-microscopic levels, increased the risk of stillbirth [26]. These findings suggest that even low-level infections that would usually be missed during routine antenatal care because they were asymptomatic and/or sub-microscopic could be detrimental to the developing fetus. The association between malaria and antepartum stillbirth was strongest when malaria was last detected and treated in the third trimester, despite screening in pregnancy; this may be the result of chronic sub-patent infections that have become patent in the third trimester or new infections in the third trimester that may be particularly harmful. In contrast, there was no strong association between malaria detected and treated in the first or second trimesters and antepartum stillbirth. However first- and second-trimester malaria (either falciparum or vivax) was strongly associated with fetal loss, principally through miscarriage, despite prompt treatment.

There are few data on the effect of confirmed malaria in pregnancy on neonatal mortality [11, 27–29], and to date, this is the largest analysis of prospective data to assess this topic. Some studies have modelled the effect of malaria in pregnancy on neonatal death through low birthweight by combining estimates of the effect of malaria (or malaria chemoprophylaxis) on birthweight, and then birthweight on neonatal death [30–32]. Such approaches assume that the effect of malaria in pregnancy on neonatal death is entirely indirect and mediated only through birthweight [27]. Traditional mediation analyses in other studies indicate that a large proportion of the effect of malaria in pregnancy on neonatal death is mediated through low birthweight and/or preterm birth [28, 29]. This is the first mediation analysis of the effect of malaria in pregnancy on stillbirth or neonatal death to account for both exposure-outcome and mediator-outcome confounders, which is a source of bias in the traditional approach to mediation analysis [33]. Additionally, by performing sequential mediation analyses of potential outcomes we overcame problems arising when there are multiple mediators and binary mediators and outcomes [22]. This is also the first analysis to quantify mediation of the effect of malaria in pregnancy on stillbirth or neonatal death through SGA. Interestingly, the proportion of the effect of malaria on neonatal death mediated through SGA and preterm was greater for vivax malaria than falciparum malaria, despite falciparum malaria more commonly being associated with these mediating variables; this may be a true effect, or it may be related to unmeasured confounding variables mentioned previously.

## Conclusions

The results of this analysis suggest that many malaria-associated stillbirths and neonatal deaths are the extreme consequences of more common adverse pregnancy outcomes caused by malaria in pregnancy. Maternal anaemia can be prevented with existing interventions including iron supplementation, infection control, and improved nutrition [34]. There is also some evidence that nutrient supplementation may counteract the impaired placental nutrient transport that is partly responsible for the effect of malaria in pregnancy on intrauterine growth restriction [35], and low-dose aspirin reduces the risk of intrauterine growth restriction [36]. Therefore, new and existing interventions in malaria-endemic settings to prevent intrauterine growth restriction and anaemia and an improved capacity for managing preterm newborns will reduce the number of malaria-associated stillbirths and neonatal deaths worldwide and augment current preventive interventions for malaria in pregnancy [8, 35]. Our results are likely an underestimation of the effects of malaria in pregnancy because of the screening and treatment programme for malaria and anaemia at SMRU. This large analysis of prospective observational data reinforces the need for malaria control programmes to focus on the prevention of malaria in pregnancy to reduce fetal loss including miscarriage, stillbirth, and neonatal death.

## Additional files

Additional file 1: Description of SMRU cohort studies with infant follow-up for the first 28 days of life. (DOCX 31 kb)

Additional file 2: Rationale for using Cox regression. (DOCX 45 kb)

Additional file 3: Selection of confounding variables and causal inference. (DOCX 180 kb)

Additional file 4: Mediation analysis: extended methods and definitions of natural direct and indirect effects. (DOCX 16 kb)

Additional file 5: Trimester of detection and symptoms of the last malaria episode detected during pregnancy. (DOCX 13 kb)

Additional file 6: The influence of recurrent malaria in pregnancy on associations between malaria and fetal loss and antepartum stillbirth. (DOCX 138 kb)

Additional file 7: Table versions of Fig. 3: The association between falciparum and vivax malaria in pregnancy and antepartum or intrapartum stillbirth. (DOCX 16 kb)

Additional file 8: Table version of Fig. 4: The association between falciparum and vivax malaria in pregnancy and fetal loss. (DOCX 13 kb)

Additional file 9: Cohort demographics of mothers in the sub-set of mother-newborn pairs followed from birth in SMRU cohort studies. (DOCX 17 kb)

Additional file 10: Discussion of potential biases. (DOCX 16 kb)

## Abbreviations

ANC: Antenatal clinic
CI: Confidence interval
EGA: Estimated gestational age
HR: Hazard ratio
SGA: Small-for-gestational-age
SMRU: Shoklo Malaria Research Unit
