# Supplementary material for: Mediation of the effect of malaria in pregnancy on stillbirth and neonatal death in an area of low transmission: observational data analysis
Source: BMC Med. 2017 May 10;15:98. doi: 10.1186/s12916-017-0863-z (PMC5424335; doi:10.1186/s12916-017-0863-z)
Supplement: Supplementary file 1 — Description of SMRU cohort studies with infant follow-up for the first 28 days of life. (DOCX 31 kb) [file 12916_2017_863_MOESM1_ESM.docx]

Additional file 1: Description of SMRU cohort studies with infant follow-up for at least the first 28 days of life

| **Primary study subject and reference to primary paper** | **Enrolment period** | **Inclusion criteria** | **Scheduled visits** | **Liveborns** | **Neonatal deaths** |
| --- | --- | --- | --- | --- | --- |
| Pharmacokinetics of amodiaquine and desethylamodiaquine in pregnant women with vivax malaria.[1] [APV] | October 2007 – November 2008 | Vivax malaria in second or third trimester and haematocrit >25%. | Monthly for six months. | 16 | 0 |
| The dynamics of pneumococcal carriage.[2] [ARI] | October 2007 – November 2008 | 28-30 weeks’ gestation | Monthly for 24 months. | 985 | 22 |
| Group B Streptococcus in pregnant women at delivery.[3] [GBS] | April 2009 – May 2010. | 28-30 weeks’ gestation | 7 days and 28 days. | 662 | 7 |
| The impact of malaria during pregnancy on infant mortality.[4] [CBx] | 1993 – 1996 | Delivered a singleton liveborn baby | Weekly for 12 months. | 1496 | 41 |
| Randomised Trial of 3 Artemisinin Combination Therapy for Malaria in Pregnancy.[5] [DMA] | February 2010 – present (ongoing) | Uncomplicated vivax or falciparum malaria | Monthly until 12 months, then every 3 months to 4 years | 335 | 1 |
| Differences in pharmacokinetics of dihydroartemisinin and piperaquine between pregnant and non-pregnant women with uncomplicated falciparum malaria.[6] [DPK] |  | Uncomplicated falciparum malaria or mixed falciparum/vivax malaria in second or third trimester and haematocrit >25%. | Monthly | 12 | 0 |
| Causes of fever in pregnant women.[7] [FPW] | May 2004 – January 2006 | Women with no fever including malaria (if not enrolled to PCA) | At one month (where possible) | 89 | 0 |
| A randomised controlled trial of artesunate-atovaquone-proguanil versus quinine for the treatment of uncomplicated multi-drug resistant falciparum malaria in pregnancy.[8] [GME] | December 2001 – July 2003 | Uncomplicated falciparum malaria in second or third trimester. | 1 week, 1 month, and then monthly until 12 months | 78 | 4 |
| The impact of micronutrient fortified flour on micronutrient status in pregnancy.[9] [NUT] | June 2004 and November 2006 | Living in Mae La refugee camp without severe anaemia and received food rations. | Fortnightly for three months, then monthly until 12 months. | 1124 | 10 |
| Assess the efficacy of artemether-lumefantrine versus artesunate for uncomplicated plasmodium falciparum treatment in pregnancy (RCT).[10] [PCA] | April 2004 – August 2006 | Uncomplicated falciparum malaria in second or third trimester. | Monthly for 12 months. | 236 | 4 |
| To determine if reported lower plasma concentrations of artemisinin derivatives for malaria in pregnancy result from reduced oral bioavailability, expanded volume of distribution or increased clearance.[11] [PWA] | April 2008 – March 2009 | Uncomplicated falciparum malaria in second or third trimester. | Monthly for 12 months. | 20 | 0 |
| Assess the efficacy of quinine plus clindamycin versus artesunate for uncomplicated plasmodium falciparum treatment in pregnancy (RCT).[12] [QAC] | October 1997 – January 2000 | Uncomplicated falciparum malaria. | 1, 3, 6, 9, and 12 months | 211 | 4 |
| Malaria in the post-partum period; a prospective cohort study.[13] [SUB] | November 2007 – January 2011 | All women attending ANC. | Every two weeks for three months, then monthly until 12 months. | 735 | 6 |
| Anthropometric markers and first year mortality in Karen refugee camps. (unpublished) [BF] | 1996 – 2002 | All liveborns. | Varied over years:  1, 2, 3, 6, 9, 12, 18, 24 months.  At one year only.  Monthly for 5 months. | 1977 | 37 |
| Effects of malaria in pregnancy on fetal growth and development in accurately dated pregnancies. (Unpublished) [UPS] | 2009 – present (ongoing) | Women with a singleton viable pregnancy between 9 and 14 weeks’ gestation. | Fortnightly until week 8, then monthly until 8 months, then at 12, 18, 24 months.  Later, follow-up changed to 1, 2, 3, 4, 6, 9, 12, 18, 24 months. | 1097 | 6 |

**References**

1. Rijken MJ, McGready R, Jullien V, Tarning J, Lindegardh N, Phyo AP, et al. Pharmacokinetics of amodiaquine and desethylamodiaquine in pregnant and postpartum women with Plasmodium vivax malaria. Antimicrob Agents Chemother. 2011;55: 4338–4342. doi:10.1128/AAC.00154-11

2. Turner P, Turner C, Jankhot A, Helen N, Lee SJ, Day NP, et al. A longitudinal study of streptococcus pneumoniae carriage in a cohort of infants and their mothers on the Thailand-Myanmar border. PLoS One. 2012;7. doi:10.1371/journal.pone.0038271

3. Turner C, Turner P, Po L, Maner N, De Zoysa A, Afshar B, et al. Group B streptococcal carriage, serotype distribution and antibiotic susceptibilities in pregnant women at the time of delivery in a refugee population on the Thai-Myanmar border. BMC Infect Dis. BioMed Central Ltd; 2012;12: 34. doi:10.1186/1471-2334-12-34

4. Luxemburger C, McGready R, Kham A, Morison L, Cho T, Chongsuphajaisiddhi T, et al. Effects of malaria during pregnancy on infant mortality in an area of low malaria transmission. Am J Epidemiol. 2001;154: 459–65.

5. McGready R. Randomised Trial of 3 Artemisinin Combination Therapy for Malaria in Pregnancy (DMA). In: ClinicalTrials.gov [Internet]. 2010 [cited 29 Jul 2016]. Available: https://clinicaltrials.gov/ct2/show/NCT01054248

6. Rijken MJ, McGready R, Phyo AP, Lindegardh N, Tarning J, Laochan N, et al. Pharmacokinetics of Dihydroartemisinin and Piperaquine in Pregnant and Nonpregnant Women with Uncomplicated Falciparum Malaria. Antimicrob Agents Chemother. 2011;55: 5500–5506. doi:10.1128/AAC.05067-11

7. Mcgready R, Ashley EA, Wuthiekanun V, Tan SO, Pimanpanarak M, Viladpai-Nguen SJ, et al. Arthropod borne disease: The leading cause of fever in pregnancy on the thai-burmese border. PLoS Negl Trop Dis. 2010;4. doi:10.1371/journal.pntd.0000888

8. McGready R, Ashley EA, Moo E, Cho T, Barends M, Hutagalung R, et al. A randomized comparison of artesunate-atovaquone-proguanil versus quinine in treatment for uncomplicated falciparum malaria during pregnancy. J Infect Dis. 2005;192: 846–53. doi:10.1086/432551

9. Stuetz W, Carrara V, Mc Gready R, Lee S, Sriprawat K, Po B, et al. Impact of Food Rations and Supplements on Micronutrient Status by Trimester of Pregnancy: Cross-Sectional Studies in the Maela Refugee Camp in Thailand. Nutrients. 2016;8: 66. doi:10.3390/nu8020066

10. McGready R, Tan SO, Ashley E a, Pimanpanarak M, Viladpai-Nguen J, Phaiphun L, et al. A randomised controlled trial of artemether-lumefantrine versus artesunate for uncomplicated plasmodium falciparum treatment in pregnancy. PLoS Med. 2008;5: e253. doi:10.1371/journal.pmed.0050253

11. McGready R, Phyo AP, Rijken MJ, Tarning J, Lindegardh N, Hanpithakpon W, et al. Artesunate/dihydroartemisinin pharmacokinetics in acute falciparum malaria in pregnancy: absorption, bioavailability, disposition and disease effects. Br J Clin Pharmacol. 2011;73: 467–477. doi:10.1111/j.1365-2125.2011.04103.x

12. Mcgready R et al. Randomized comparison of quinine-clindamycin treatment of falciparum malaria in pregnancy versus attesunate in the. Trans R Soc Trop Med Hyg. 2001;95: 651–656. doi:10.1016/S0035-9203(01)90106-3

13. Boel ME, Rijken MJ, Leenstra T, Pyae Phyo A, Pimanpanarak M, Keereecharoen NL, et al. Malaria in the post-partum period; a prospective cohort study. PLoS One. 2013;8: 8–13. doi:10.1371/journal.pone.0057890
