# Supplementary material for: Mediation of the effect of malaria in pregnancy on stillbirth and neonatal death in an area of low transmission: observational data analysis
Source: BMC Med. 2017 May 10;15:98. doi: 10.1186/s12916-017-0863-z (PMC5424335; doi:10.1186/s12916-017-0863-z)
Supplement: Supplementary file 2 — Rationale for using Cox regression. (DOCX 45 kb) [file 12916_2017_863_MOESM2_ESM.docx]

**Additional file 2: Rationale for using Cox regression**

Cox regression was chosen to account for variable follow-up times between women (from the first antenatal clinic visit until birth), left-truncation (see below), lost-to-follow-up, time-varying malaria status (as women are screened throughout pregnancy), and time-varying risk of stillbirth, fetal death, and neonatal death (see Figure).

Our data is left truncated[1,2] because fetal death (either miscarriage or stillbirth) may occur before the woman ever presents for antenatal care. That is, observation does not begin until sometime after women become at risk of the outcome. Additionally, the risk of fetal death changes as a pregnancy progresses. Therefore, entry into the analysis is differential by the outcome (fetal death), and also by the exposure (those with malaria are prompted to present earlier). This will induce bias unless left truncation is accounted for using survival analysis with gestation time as the time scale, and only allowing women to enter the analysis when they come under observation (i.e. at the first antenatal consultation), rather than when they became at risk of fetal death (i.e. at conception).

Figure. Smoothed hazard of antepartum stillbirth, neonatal death, and fetal loss over analysis time.

1. Howards PP, Hertz-Picciotto I, Poole C. Conditions for bias from differential left truncation. Am J Epidemiol. 2006;165: 444–452.

2. Meister R, Schaefer C. Statistical methods for estimating the probability of spontaneous abortion in observational studies-analyzing pregnancies exposed to coumarin derivatives. Reprod Toxicol. 2008;26: 31–5. doi:10.1016/j.reprotox.2008.06.006
