# Supplementary material for: Mediation of the effect of malaria in pregnancy on stillbirth and neonatal death in an area of low transmission: observational data analysis
Source: BMC Med. 2017 May 10;15:98. doi: 10.1186/s12916-017-0863-z (PMC5424335; doi:10.1186/s12916-017-0863-z)
Supplement: Supplementary file 3 — Selection of confounding variables and causal inference. (DOCX 180 kb) [file 12916_2017_863_MOESM3_ESM.docx]

**Additional file 3: Selection of confounding variables and causal inference**

Confounding variables of the association between malaria in pregnancy and adverse outcomes were selected by drawing a directed acyclic graph in DAGitty v2.3 (dagitty.net) (Figure).[1] The inference that malaria in pregnancy has a causal effect on adverse outcomes (stillbirth and neonatal death) is dependent on the assumption that unmeasured variables are not confounding the association. Data on smoking was not routinely collected until 1997, and could therefore not be adjusted for. The proportion of women who smoke is higher in women with malaria in pregnancy (34%) than women with no malaria in pregnancy (22%) because the smokers are the old traditional Karen women, who traditionally go to the forest to get food (and malaria).

Figure. Directed acyclic graph for the association between malaria in pregnancy and adverse birth outcomes (stillbirth, fetal death, or neonatal death). Yellow: exposure; blue: outcome; white: adjusted confounder; grey: unmeasured variable; green: causal path; red: biasing path. Maternal age was omitted due to collinearity with gravidity. Potential unmeasured confounders include socio-economic status (SES), education, occupation, migration, and environmental factors.

1. Textor J, Hardt J, Knüppel S. DAGitty: A graphical tool for analyzing causal diagrams. Epidemiology. 2011;5: 745.
