# Supplementary material for: Mediation of the effect of malaria in pregnancy on stillbirth and neonatal death in an area of low transmission: observational data analysis
Source: BMC Med. 2017 May 10;15:98. doi: 10.1186/s12916-017-0863-z (PMC5424335; doi:10.1186/s12916-017-0863-z)
Supplement: Supplementary file 4 — Mediation analysis: extended methods and definitions of natural direct and indirect effects. (DOCX 16 kb) [file 12916_2017_863_MOESM4_ESM.docx]

**Additional file 4: Mediation analysis – extended methods and definitions of natural direct and indirect effects.**

- We used a sequential approach because there were multiple mediators that are not independent of each other (Figure 1 of manuscript).
- Inverse probability weights were calculated from predicted probabilities of malaria derived from a logistic regression analysis of the potential confounders (gravidity, clinic site, and yearly malaria incidence) and malaria. The weights were calculated in order to achieve balance in terms of the confounders between those individuals with and without malaria.
- Potential outcomes (i.e. the expected outcome [for stillbirth, fetal death, or neonatal death] given the values of the exposure and mediators) were estimated from logistic models, and expressed as the risk of the birth outcome by converting the predicted odds of the birth outcome to a risk using the formula: risk = odds/(1+odds). Potential outcomes were weighted by the inverse probability of malaria (see previous point).
- Potential outcomes were estimated in the sequence below, because of the associations between the mediators (Figure 1 of manuscript). We have used the following notation for the potential outcomes at given levels of the exposure (malaria) and mediators: E{Y(a, M_1_(a’), M_2_(a’’))}, where Y is the birth outcome, a is malaria (0 for unexposed and 1 for exposed), M_1_ is anaemia or preterm birth, and M_2_ is SGA. Where the birth outcome was stillbirth or fetal death, M_1_ was maternal anaemia and M_2_ was SGA (preterm birth is a collider rather than a mediator so was not considered). Where the birth outcome was neonatal death, M_1_ was SGA and M_2_ was preterm birth (maternal anaemia was not considered because <1% of women in this sub-set were anaemic). The marginal natural direct effects (NDE) and marginal natural indirect effects (NIEs) were expressed as risk ratios of potential outcomes. The values of the mediators, a’ and a’’, are 0 when the mediator is set to it’s natural value under no exposure to malaria, and 1 when the mediator is allowed to change to what it would be if all women were exposed to malaria from what it would be if there had been no exposure to malaria.

1. We first fit a multivariable logistic model for the outcome with the covariates malaria, both mediators, and confounders, to obtain the marginal NDE: E{Y(1, M_1_(0), M_2_(0))} / E{Y(0, M_1_(0), M_2_(0))}. The marginal NDE is the relative change in the risk of the birth outcome if every woman was exposed to malaria versus if every woman was unexposed to malaria, if the risk of both mediators were set to their natural levels under no exposure to malaria.
2. We then fit the same logistic model, excluding M_2_, to obtain the marginal NDE and marginal NIE allowing for mediation through M_2_, but not M_1_.

- NDE: E{Y(1, M_1_(0), M_2_(1))} / E{Y(0, M_1_(0), M_2_(1))}. The marginal NDE when mediation is allowed through M_2_, but not M_1_, is the relative change in the risk of the birth outcome if every woman was exposed to malaria versus if every women was unexposed to malaria, if the risk of M_1_ was set to its natural level under no exposure to malaria.
- NIE: E{Y(1, M_1_(0), M_2_(1))} / E{Y(1, M_1_(0), M_2_(0))}. The marginal NIE through M_2_, but not M_1_, is the relative change in the risk of the birth outcome if the risk of M_2_ is allowed to change to what it would be if all women were exposed to malaria from what it would be if there had been no exposure to malaria, and the risk of M_1_ was set to its natural levels under no exposure to malaria.

1. Finally, M_1_ was also removed from the logistic model to obtain the marginal NDE and marginal NIE allowing for mediation through M_1_ (and M_2_).

- NDE: E{Y(1, M_1_(1), M_2_(1))} / E{Y(0, M_1_(1), M_2_(1))}. The marginal NDE when mediation is allowed through all mediators is the relative change in the risk of the birth outcome if every woman was exposed to malaria compared to if every women was unexposed to malaria.
- NIE: E{Y(1, M_1_(1), M_2_(1))} / E{Y(1, M_1_(0), M_2_(0))}. The marginal NIE through M_2_ and M_1_ is the relative change in the risk of the birth outcome if the risk of M_1_ (and M_2_) are allowed to change to what it would be if all women were exposed to malaria from what it would be if there was no exposure to malaria.
- 95% confidence intervals for marginal NDE and NIE risk ratios were obtained by bootstrapping using the percentile method and 1000 replications.
