# Supplementary material for: Mediation of the effect of malaria in pregnancy on stillbirth and neonatal death in an area of low transmission: observational data analysis
Source: BMC Med. 2017 May 10;15:98. doi: 10.1186/s12916-017-0863-z (PMC5424335; doi:10.1186/s12916-017-0863-z)
Supplement: Supplementary file 5 — Trimester of detection and symptoms of the last malaria episode detected during pregnancy. (DOCX 13 kb) [file 12916_2017_863_MOESM5_ESM.docx]

Additional file 5: Trimester of detection and symptoms of the last malaria episode detected during pregnancy

| **Trimester of detection** | **Asymptomatic** | **Symptomatic** |
| --- | --- | --- |
| **Falciparum malaria** |  |  |
| First | 169 (24) | 546 (76) |
| Second | 630 (45) | 781 (55) |
| Third | 636 (47) | 716 (53) |
| **Vivax malaria** |  |  |
| First | 301 (46) | 353 (54) |
| Second | 886 (65) | 479 (35) |
| Third | 1491 (66) | 764 (34) |

*p-*value <0.001 for the association between trimester of detection and symptoms for both falciparum and vivax malaria.
