# Supplementary material for: Mediation of the effect of malaria in pregnancy on stillbirth and neonatal death in an area of low transmission: observational data analysis
Source: BMC Med. 2017 May 10;15:98. doi: 10.1186/s12916-017-0863-z (PMC5424335; doi:10.1186/s12916-017-0863-z)
Supplement: Supplementary file 6 — The influence of recurrent malaria in pregnancy on associations between malaria and fetal loss and antepartum stillbirth. (DOCX 138 kb) [file 12916_2017_863_MOESM6_ESM.docx]

**Additional file 6: The influence of recurrent malaria in pregnancy on associations between malaria and fetal loss and antepartum stillbirth.**

Table 1. The association between initial and recurrent malaria in pregnancy and fetal loss and antepartum stillbirth

|  | **Fetal loss** | | **Miscarriage** | | **Antepartum stillbirth** | |
| --- | --- | --- | --- | --- | --- | --- |
|  | **Adjusted HR [95% CI]** | **n/N** | **Adjusted HR [95% CI]** | **n/N** | **Adjusted HR [95% CI]** | **n/N** |
| **Falciparum malaria** |  |  |  |  |  |  |
| No malaria | Reference group | 5246/52486 | Reference group | 4719/51983 | Reference group | 202/52343 |
| Initial episode | 1.75 [1.52, 2.01] | 222/2586 | 1.74 [1.49, 2.04] | 177/2549 | 2.26 [1.41, 3.62] | 21/2571 |
| Recurrent episode | 2.65 [2.02, 3.49] | 54/892 | 3.18 [2.31, 4.36] | 40/884 | 2.15 [0.99, 4.67] | 7/890 |
| **Vivax malaria** |  |  |  |  |  |  |
| No malaria | Reference group | 5246/52486 | Reference group | 4719/51983 | Reference group | 202/52343 |
| Initial episode | 1.17 [1.02, 1.35] | 224/2804 | 1.21 [1.05, 1.40] | 201/2788 | 1.07 [0.57, 2.04] | 10/2799 |
| Recurrent episode | 1.51 [1.12, 2.05] | 43/1470 | 1.79 [1.24, 2.58] | 29/1463 | 1.00 [0.41, 2.44] | 5/1469 |

The reference group refers to women without falciparum malaria or vivax malaria in pregnancy. Malaria in pregnancy was a time-dependent variable. Models were adjusted for gravidity, clinic site, and yearly malaria incidence. Fetal loss includes miscarriages and stillbirths. Miscarriage refers to fetal loss before 28 weeks’ gestation.

Figure. The association between malaria in pregnancy and antepartum stillbirth excluding women with recurrent malaria.
