# Supplementary material for: Mediation of the effect of malaria in pregnancy on stillbirth and neonatal death in an area of low transmission: observational data analysis
Source: BMC Med. 2017 May 10;15:98. doi: 10.1186/s12916-017-0863-z (PMC5424335; doi:10.1186/s12916-017-0863-z)
Supplement: Supplementary file 7 — Table versions of Fig. 3: The association between falciparum and vivax malaria in pregnancy and antepartum or intrapartum stillbirth. (DOCX 16 kb) [file 12916_2017_863_MOESM7_ESM.docx]

Additional file 7: Table versions of Figure 3

Table 1: The association between falciparum and vivax malaria in pregnancy and antepartum stillbirth

|  | **Unadjusted HR [95% CI]; *p*-value** | **Adjusted HR [95% CI]; *p*-value** |
| --- | --- | --- |
| **Falciparum malaria** |  |  |
| Falciparum malaria (all) | Reference Group | Reference Group |
|  | 2.32 [1.56, 3.44]; <0.001 | 2.24 [1.47, 3.41]; <0.001 |
| Asymptomatic falciparum | Reference Group | Reference Group |
|  | 1.42 [0.67, 3.01]; 0.364 | 1.35 [0.61, 2.96]; 0.459 |
| Symptomatic falciparum | Reference Group | Reference Group |
|  | 3.06 [1.89, 4.96]; <0.001 | 2.99 [1.83, 4.89]; <0.001 |
| Trimester | Reference Group | Reference Group |
| First | Not estimated | Not estimated |
| Second | 1.62 [0.86, 3.05]; 0.139 | 1.59 [0.83, 3.04]; 0.166 |
| Third | 4.44 [2.73, 7.23]; <0.001 | 4.33 [2.58, 7.25]; <0.001 |
| **Vivax malaria** |  |  |
| Vivax malaria (all) | Reference Group | Reference Group |
|  | 0.97 [0.57, 1.64]; 0.905 | 1.05 [0.61, 1.79]; 0.868 |
| Asymptomatic vivax | Reference Group | Reference Group |
|  | 0.49 [0.18, 1.32]; 0.158 | 0.54 [0.20, 1.45]; 0.219 |
| Symptomatic vivax | Reference Group | Reference Group |
|  | 2.10 [1.08, 4.09]; 0.030 | 2.21 [1.12, 4.33]; 0.021 |
| Trimester | Reference Group | Reference Group |
| First | 0.58 [0.08, 4.13]; 0.586 | 0.60 [0.08, 4.32]; 0.616 |
| Second | 0.43 [0.14, 1.34]; 0.144 | 0.46 [0.15, 1.45]; 0.187 |
| Third | 1.64 [0.89, 3.02]; 0.113 | 1.79 [0.96, 3.34]; 0.066 |

The reference group refers to women without falciparum malaria or vivax malaria in pregnancy. Models were adjusted for gravidity, clinic site, and yearly malaria incidence.

Table 2: The association between falciparum and vivax malaria in pregnancy and intrapartum stillbirth

|  | **Unadjusted HR [95% CI]; *p*-value** | **Adjusted HR [95% CI]; *p*-value** |
| --- | --- | --- |
| **Falciparum malaria** |  |  |
| Falciparum malaria (all) | Reference Group | Reference Group |
|  | 1.55 [0.90, 2.69]; 0.117 | 1.03 [0.58, 1.83]; 0.910 |
| Asymptomatic falciparum | Reference Group | Reference Group |
|  | 1.83 [0.86, 3.92]; 0.118 | 1.01 [0.46, 2.24]; 0.975 |
| Symptomatic falciparum | Reference Group | Reference Group |
|  | 1.39 [0.61, 3.14]; 0.430 | 1.12 [0.49, 2.54]; 0.795 |
| Trimester | Reference Group | Reference Group |
| First | 2.36 [0.75, 7.39]; 0.142 | 1.96 [0.62, 6.17]; 0.249 |
| Second | 1.17 [0.48, 2.86]; 0.724 | 0.76 [0.31, 1.88]; 0.549 |
| Third |  |  |
| **Vivax malaria** | 1.72 [0.76, 3.90]; 0.194 | 1.09 [0.47, 2.52]; 0.839 |
| Vivax malaria (all) | Reference Group | Reference Group |
|  | 1.12 [0.64, 1.98]; 0.686 | 1.18 [0.66, 2.11]; 0.574 |
| Asymptomatic vivax | Reference Group | Reference Group |
|  | 0.65 [0.24, 1.75]; 0.389 | 0.70 [0.26, 1.90]; 0.482 |
| Symptomatic vivax | Reference Group | Reference Group |
|  | 1.55 [0.63, 3.77]; 0.338 | 1.61 [0.65, 3.94]; 0.301 |
| Trimester | Reference Group | Reference Group |
| First | 0.85 [0.12, 6.05]; 0.867 | 0.85 [0.12, 6.06]; 0.868 |
| Second | 0.68 [0.22, 2.12]; 0.503 | 0.71 [0.23, 2.24]; 0.561 |
| Third | 1.52 [0.77, 2.98]; 0.228 | 1.61 [0.81, 3.21]; 0.173 |

The reference group refers to women without falciparum malaria or vivax malaria in pregnancy. Models were adjusted for gravidity, clinic site, and yearly malaria incidence.
