# Supplementary material for: Mediation of the effect of malaria in pregnancy on stillbirth and neonatal death in an area of low transmission: observational data analysis
Source: BMC Med. 2017 May 10;15:98. doi: 10.1186/s12916-017-0863-z (PMC5424335; doi:10.1186/s12916-017-0863-z)
Supplement: Supplementary file 8 — Table version of Fig. 4: The association between falciparum and vivax malaria in pregnancy and fetal loss. (DOCX 13 kb) [file 12916_2017_863_MOESM8_ESM.docx]

Additional file 8: Table version of Figure 4 ‘The association between falciparum and vivax malaria in pregnancy and fetal loss’

|  | **Unadjusted HR [95% CI]; *p*-value** | **Adjusted HR [95% CI]; *p*-value** |
| --- | --- | --- |
| **Falciparum malaria** |  |  |
| Falciparum malaria (all) | Reference Group | Reference Group |
|  | 1.70 [1.51, 1.93]; <0.001 | 1.87 [1.65, 2.12]; <0.001 |
| Asymptomatic falciparum | Reference Group | Reference Group |
|  | 1.34 [1.03, 1.74]; 0.028 | 1.54 [1.18, 2.00]; 0.001 |
| Symptomatic falciparum | Reference Group | Reference Group |
|  | 2.09 [1.82, 2.40]; <0.001 | 2.20 [1.91, 2.54]; <0.001 |
| Trimester | Reference Group | Reference Group |
| First | 1.61 [1.38, 1.88]; <0.001 | 1.73 [1.48, 2.02]; <0.001 |
| Second | 1.68 [1.32, 2.14]; <0.001 | 1.95 [1.53, 2.49]; <0.001 |
| Third | 2.73 [1.89, 3.95]; <0.001 | 3.12 [2.16, 4.53]; <0.001 |
| **Vivax malaria** |  |  |
| Vivax malaria (all) | Reference Group | Reference Group |
|  | 1.09 [0.96, 1.23]; 0.173 | 1.22 [1.07, 1.38]; 0.002 |
| Asymptomatic vivax | Reference Group | Reference Group |
|  | 1.13 [0.94, 1.35]; 0.195 | 1.23 [1.02, 1.47]; 0.027 |
| Symptomatic vivax | Reference Group | Reference Group |
|  | 1.78 [1.50, 2.13]; <0.001 | 1.92 [1.61, 2.30]; <0.001 |
| Trimester | Reference Group | Reference Group |
| First | 1.05 [0.90, 1.22]; 0.535 | 1.18 [1.02, 1.37]; 0.030 |
| Second | 1.10 [0.85, 1.43]; 0.465 | 1.21 [0.93, 1.57]; 0.154 |
| Third | 1.47 [0.99, 2.19]; 0.056 | 1.60 [1.08, 2.38]; 0.020 |

The reference group refers to women without falciparum malaria or vivax malaria in pregnancy. Models were adjusted for gravidity, clinic site, and yearly malaria incidence.
