# Supplementary material for: Mediation of the effect of malaria in pregnancy on stillbirth and neonatal death in an area of low transmission: observational data analysis
Source: BMC Med. 2017 May 10;15:98. doi: 10.1186/s12916-017-0863-z (PMC5424335; doi:10.1186/s12916-017-0863-z)
Supplement: Supplementary file 9 — Cohort demographics of mothers in the sub-set of mother-newborn pairs followed from birth in SMRU cohort studies. (DOCX 17 kb) [file 12916_2017_863_MOESM9_ESM.docx]

Additional file 9: Cohort demographics of mothers in the sub-set of mother-newborn pairs followed from birth in SMRU cohort studies

Table 1. Cohort demographics of mothers in the sub-set of mother-newborn pairs followed from birth in SMRU cohort studies, by malaria in pregnancy, N = 9090

| **Variable** | **No malaria, N = 6623** | **Malaria, N = 2467** |
| --- | --- | --- |
| EGA at first ANC (start of follow-up), weeks | 11.3 {8.2, 17.0}, 0.1 – 41.0 | 13.0 {9.0, 19.7}, 0.1 – 39.8 |
| EGA method |  |  |
| Ultrasound biometry | 4590 (69) | 1181 (48) |
| Dubowitz | 1732 (26) | 974 (39) |
| Fundal Height | 235 (4) | 262 (11) |
| Last menstrual period | 66 (1) | 51 (2) |
| Maternal age, years | 25 {21, 30}, 14 – 48 | 24 {20, 30}, 14 – 47 |
| Primigravid† | 1681 (25) | 716 (29) |
| Haematocrit†*, % | 33 {31, 36}, 18 – 47 | 33 {30, 35}, 16 – 48 |
| Anaemia†* | 6 (0) | 16 (1) |
| Current smoker† | 1049 (22) | 599 (35) |
| Site |  |  |
| Refugee camp | 5235 (79) | 1283 (52) |
| Migrant community | 1388 (21) | 1185 (48) |
| Malaria in pregnancy | - | 2467 (100) |
| Falciparum malaria only | - | 904 (37) |
| Vivax malaria only | - | 985 (40) |
| Falciparum and vivax malaria** | - | 578 (23) |
| Small-for-gestational-age† | 1509 (25) | 664 (33) |
| Preterm birth | 572 (9) | 282 (11) |
| Neonatal death | 94 (1) | 50 (2) |
| Lost to follow-up*** | 2035 (31) | 969 (39) |
| Day last seen | 0 {0, 0}, 0 – 27 | 0 {0, 1}, 0 – 27 |

EGA: estimated gestational age; ANC: antenatal clinic. Numbers are median {IQR}, range or frequency (%). †Missing data: gravidity 2 [0%] (1 [0%] with no malaria; 1 [0%] with malaria); smoking status 2514 [28%] (1770 [27%] with no malaria; 744 [30%] with malaria); haematocrit 153 [2%] (147 [2%] with no malaria; 6 [0%] with malaria); small-for-gestational-age 178 [2%] (103 [2%] with no malaria; 75 [3%] with malaria). *Last measurement during pregnancy; anaemia defined as last haematocrit measurement during pregnancy <30%. **Women with either a mixed infection or multiple infections of different species. ***Mothers of newborns lost to follow-up before 28 days were more likely to have malaria and smoke.

Table 2. Cohort demographics of mothers in the sub-set of mother-newborn pairs followed from birth in SMRU cohort studies, by lost to follow-up, N = 9090

| **Variable** | **Retained, N = 6086** | **Lost*, N = 3004** |
| --- | --- | --- |
| EGA at first ANC (start of follow-up), weeks | 12.9 {9, 20.3}, 0.1-41 | 11.2 {8.1, 16.5}, 0.1-39.8 |
| Maternal age, years | 25 {20, 30}, 15-48 | 25 {21, 30}, 14-48 |
| Primigravid† | 1602 (26) | 794 (26) |
| Haematocrit†**, % | 33 {30, 35}, 17-48 | 33 {31, 36}, 16-47 |
| Anaemia†** | 8 (0) | 14 (0) |
| Current smoker† | 1235 (24) | 413 (31) |
| Site |  |  |
| Refugee camp | 4227 (69) | 2290 (76) |
| Migrant community | 1859 (31) | 714 (24) |
| Small-for-gestational-age† | 1322 (22) | 785 (28) |
| Preterm birth | 453 (7) | 401 (13) |

*Lost to follow-up before 28 days of life. EGA: estimated gestational age; ANC: antenatal clinic. Numbers are median {IQR}, range or frequency (%). †Missing data: gravidity 2 [0%]; smoking status 2514 [28%]; haematocrit 153 [2%]; small-for-gestational-age 178 [2%]. **Last measurement during pregnancy; anaemia defined as last haematocrit measurement during pregnancy <30%.
