# Supplementary material for: Mediation of the effect of malaria in pregnancy on stillbirth and neonatal death in an area of low transmission: observational data analysis
Source: BMC Med. 2017 May 10;15:98. doi: 10.1186/s12916-017-0863-z (PMC5424335; doi:10.1186/s12916-017-0863-z)
Supplement: Supplementary file 10 — Discussion of potential biases. (DOCX 16 kb) [file 12916_2017_863_MOESM10_ESM.docx]

**Additional file 10: Discussion of potential biases.**

***Bias due to varying methods of estimating gestational age:***

Women with malaria were more likely to have had their gestational age estimated from symphysis fundal height measurements, which overestimates gestational age (especially in preterm newborns) compared to ultrasound biometry.[1] Additionally, the hazard of stillbirth increases with gestation (Supplementary Figure 1), and Cox regression models account for this by comparing women of the same estimated gestational age at the time of stillbirth. Therefore, differences in the method of estimating gestational age will have underestimated associations between malaria and stillbirth because the true gestational age of women with malaria is probably slightly younger than their estimated gestational age, which is arguably more tolerable than an overestimation (whereby it would be impossible to know if there was a true association). Bias due to potential non-differential misclassification of late miscarriages as stillbirths (as gestation is recorded as the time of delivery rather than the time of death) between women with and without malaria in pregnancy is also possible.[2] Misclassification of mediating variables (SGA and preterm birth) will underestimate the proportion of the total effect that is mediated.[3]

***Bias due to informative right censoring:***

Cox regression assumes non-informative censoring, but it is possible that there was an association between death and loss to follow-up (i.e. informative right censoring). This is likely to be less of a problem in the analysis of stillbirth than in the analysis of all fetal loss, since women who miscarry may not return to antenatal care, especially if they miscarry early in pregnancy. Informative right censoring is more likely in the neonatal death analysis, as newborns who were lost to follow up were more likely to have been small for gestational age or preterm, and to have been born to mothers who had malaria in pregnancy (Additional file 9). If censoring was informative, the assumption of non-informative censoring will have resulted in an underestimation the associations.

1. Moore KA, Simpson JA, Thomas KH, Rijken MJ, White LJ, Dwell SLM, et al. Estimating gestational age in late presenters to antenatal care in a resource-limited setting on the Thai-Myanmar border. PLoS One. 2015;10: e0131025. doi:10.1371/journal.pone.0131025

2. Gullen WH, Bearman JE, Johnson EA. Effects of misclassification in epidemiologic studies. Public Health Rep. 1968;83: 914–918.

3. Ogburn EL, VanderWeele TJ. On the Nondifferential Misclassification of a Binary Confounder. Epidemiology. 2012;23: 433–439. doi:10.1097/EDE.0b013e31824d1f63
